# Regulation of the Regulators: Post-Translational Modifications, Subcellular, and Spatiotemporal Distribution of Plant 14-3-3 Proteins

**DOI:** 10.3389/fpls.2016.00611

**Published:** 2016-05-09

**Authors:** Rashaun S. Wilson, Kirby N. Swatek, Jay J. Thelen

**Affiliations:** Department of Biochemistry, Christopher S. Bond Life Sciences Center, University of MissouriColumbia, MO, USA

**Keywords:** 14-3-3, phosphorylation, plants, subcellular localization, post-translational modifications

## Abstract

14-3-3 proteins bind to and modulate the activity of phosphorylated proteins that regulate a variety of metabolic processes in eukaryotes. Multiple 14-3-3 isoforms are expressed in most organisms and display redundancy in both sequence and function. Plants contain the largest number of 14-3-3 isoforms. For example, *Arabidopsis thaliana* contains thirteen 14-3-3 genes, each of which is expressed. Interest in the plant 14-3-3 field has swelled over the past decade, largely due to the vast number of possibilities for 14-3-3 metabolic regulation. As the field progresses, it is essential to understand these proteins' activities at both the spatiotemporal and subcellular levels. This review summarizes current knowledge of 14-3-3 proteins in plants, including 14-3-3 interactions, regulatory functions, isoform specificity, and post-translational modifications. We begin with a historical overview and structural analysis of 14-3-3 proteins, which describes the basic principles of 14-3-3 function, and then discuss interactions and regulatory effects of plant 14-3-3 proteins in specific tissues and subcellular compartments. We conclude with a summary of 14-3-3 phosphorylation and current knowledge of the functional effects of this modification in plants.

## Introduction

The 14-3-3 proteins exist in all eukaryotes and regulate protein function generally through a direct association with phosphorylated proteins. These small, acidic proteins can function as homo- or hetero-dimers, and each monomer is capable of interacting with phosphorylated serine or threonine residues of binding partners. This structural characteristic allows 14-3-3 proteins to act as scaffolding molecules during signal transduction events, bringing two phosphoproteins within close proximity of one another. Of all eukaryotes, plants contain the largest number of 14-3-3 gene paralogs, which increases the combinatorial possibilities for specialized roles. Plants have developed fine-tuned mechanisms for cellular control through protein-protein interactions. 14-3-3 proteins are capable of influencing their binding partners in various ways, including their subcellular localization and enzymatic activities, which can vary in different organs, from primary metabolism to phototropism. Here, we focus on three emerging areas of 14-3-3 regulation: subcellular compartmentalization, spatiotemporal expression, and post-translational modification.

### Evolutionary background of 14-3-3 proteins

14-3-3 proteins are ancient, eukaryotic phosphobinding proteins. The discrete arginine and lysine residues that interact with the phosphate group of 14-3-3 binding partners are conserved across eukaryotes. Membrane-bound subcellular organelles are unique to eukaryotes, and 14-3-3 proteins affect binding partners within these compartments. Cytoplasmic sequestration of transcription factors, activation of mitochondrial enzymes, and guidance of chloroplast precursor proteins are a few examples of how 14-3-3 proteins influence these organelles. It is also evident that 14-3-3 proteins are essential to eukaryotic function. For example, disruption of the two 14-3-3 genes (BMH1, BMH2) in yeast was reported to be lethal, but the introduction of an *Arabidopsis* 14-3-3 protein rescued this phenotype (van Heusden et al., 1995), suggesting a conserved functional role between plant and fungal 14-3-3 proteins.

### Dimerization and phosphobinding partner recognition

**Figure 1:**
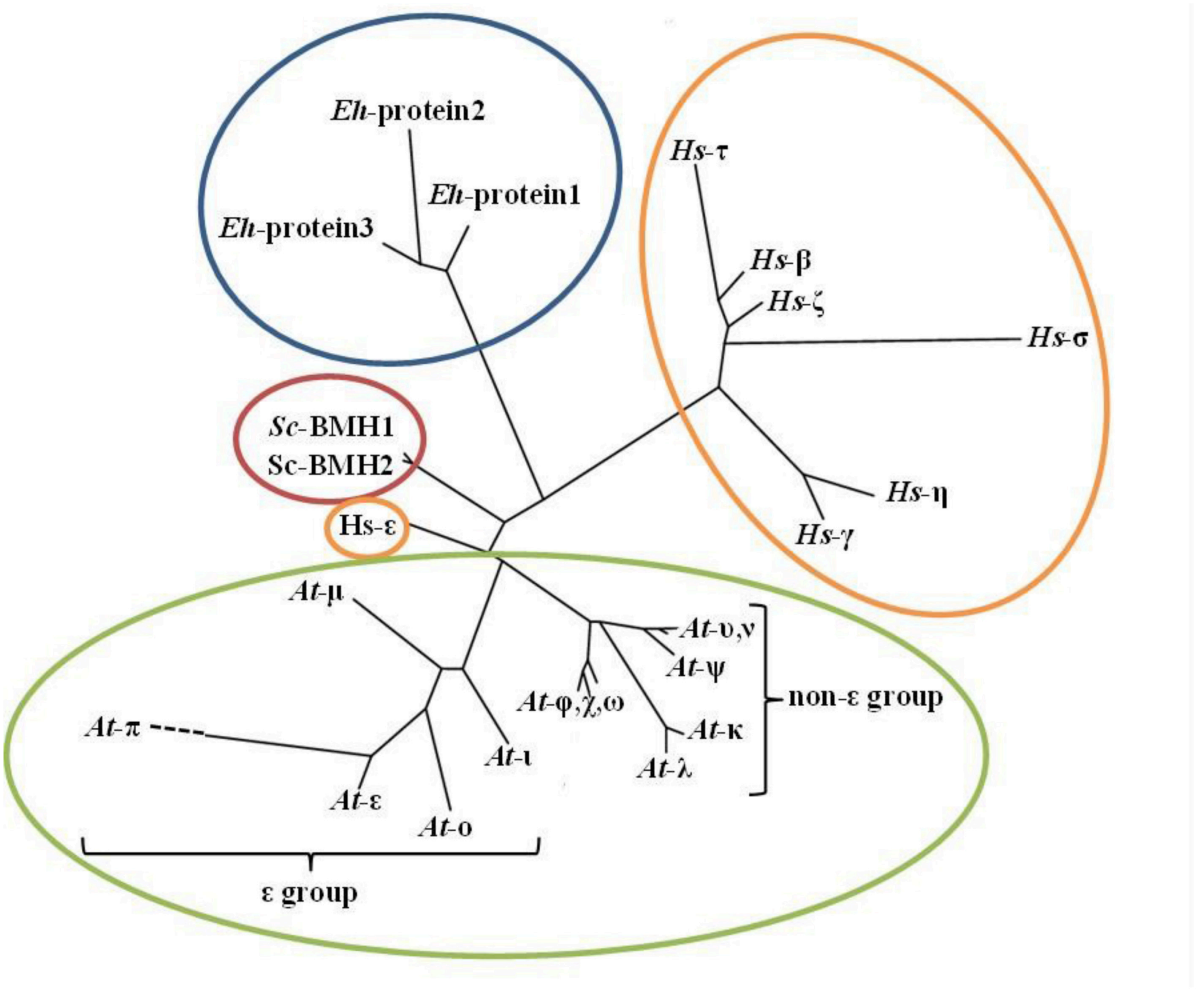
**Phylogenetic tree of 14-3-3 paralogs from eukaryotic species**. The depicted species are (1) Plantae; *Arabidopsis thaliana* (mouse-ear cress), (2) Animalia; *Homo sapiens* (human), (3) Fungi; *Saccharomyces cerevisiae* (baker's yeast), (4) Protista; *Entamoeba histolytica. Arabidopsis* 14-3-3 isoforms are divided into two distinct subgroups: epsilon (ε) and non-epsilon. The phylogenetic tree was generated with the Phylogeny.fr program (http://www.phylogeny.fr/) tree-style phylogram, using the full-length 14-3-3 protein sequences from each species.

All eukaryotes possess multiple 14-3-3 gene paralogs, adding functional complexity to this family of regulatory proteins. This multigenic complexity has many genomic, molecular, and biochemical characteristics, including (1) a broad range of distinct gene paralogs among eukaryotes; (2) variable spatiotemporal expression among paralogs; (3) diversity of subcellular localization; (4) variable binding partner motifs; and (5) variable 14-3-3 dimerization domains. 14-3-3 proteins interact with their binding partners through three well-defined binding motifs, which upon phosphorylation act as a target for 14-3-3 binding. These canonical 14-3-3 binding motifs include mode I—RXX(pS/pT)XP (Yaffe et al., 1997), mode II—RX(F/Y)X(pS)XP (Yaffe et al., 1997), and mode III—SW(pT)X-COOH (Coblitz et al., 2005). A modified mode I motif, LX(R/K)SX(pS/pT)XP, is prevalent in plants (Johnson et al., 2010). In *Arabidopsis*, 13 unique paralogs exist and can be subdivided into two distinct subgroups, epsilon and non-epsilon (Figure 1). These paralogs allow for the formation of up to 169 dimers (both homo- and hetero-dimers), and this variation is believed to increase their functional diversity. Mammals generally contain seven 14-3-3 paralogs, while fungi usually contain two (Figure 1). Multiple studies have assessed the binding partner specificity of 14-3-3 gene paralogs and have found, in general, moderate changes in binding partner preference rather than an abrupt inability to interact (Lambeck et al., 2010), suggesting a high level of functional overlap among plant paralogs. This finding could explain how a plant 14-3-3 protein was able to rescue a lethal phenotype in yeast 14-3-3 knockout lines (van Heusden et al., 1995). This functional redundancy is also supported by 14-3-3 sequence conservation that exists across isoforms of many species. Variation in 14-3-3 sequence primarily occurs at the N- and C-termini, which are implicated in dimerization and binding partner entry/exit, respectively, and are thought to contribute to 14-3-3 isoform specificity (Ferl et al., 1994; Pallucca et al., 2014).

### Subcellular dynamics and functions of plant 14-3-3 proteins

#### Cytoplasm

**Figure 2:**
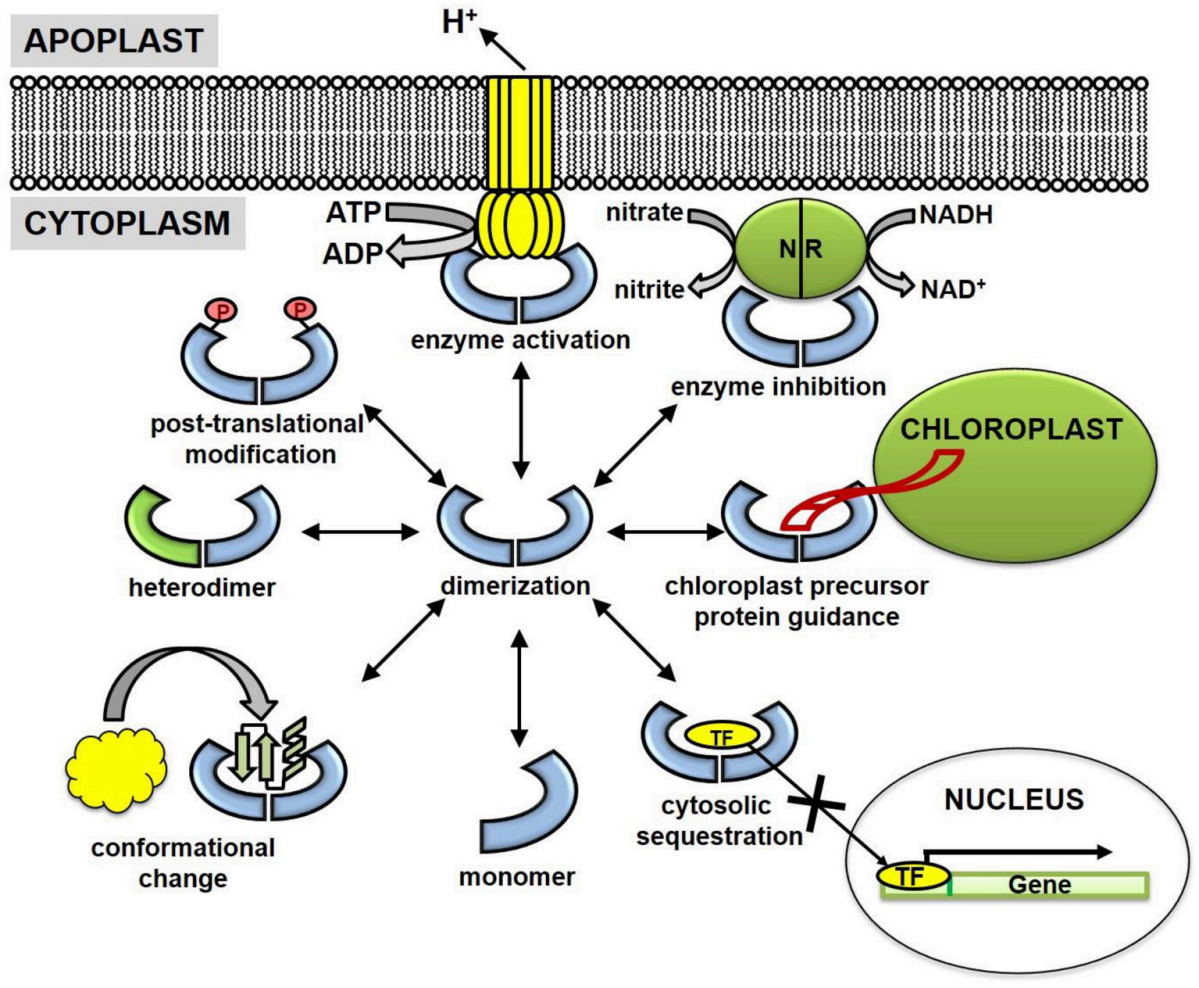
**Cellular mechanisms of plant 14-3-3 proteins**. 14-3-3 proteins are generally believed to function as dimers (hetero- and homo-dimers), although evidence also supports a functional role for 14-3-3 monomers. Several key 14-3-3 mechanisms are enzyme activation, enzyme inhibition, chloroplast precursor protein guidance, cytosolic sequestration, and conformational changes of binding partners. Post-translational modifications (PTMs) of 14-3-3 proteins (phosphorylation, acetylation, ubiquitination) have been identified but lack the functional characterization. NR, nitrate reductase and TF, transcription factor.

Initial research suggested 14-3-3 proteins were primarily cytosolic. However, subsequent research has detected 14-3-3 proteins in nearly every other subcellular compartment, including the nucleus (Taoka et al., 2011), plastid (Sehnke et al., 2001), cell membrane (Swatek et al., 2014), and mitochondria (Bunney et al., 2001). Nevertheless, binding and subsequent cytoplasmic sequestration of transcription factors is one well-characterized regulatory function of 14-3-3 proteins (Figure 2). For example, upon phosphorylation, transcription factor BZR1 and a bZIP transcriptional activator RSG are retained in the cytoplasm through binding of 14-3-3 λ, ω, and μ (Igarashi et al., 2001; Ishida et al., 2004; Gampala et al., 2007). Site-directed mutagenesis of the canonical 14-3-3 binding motif prevented trafficking and resulted in strong nuclear localization of these transcription factors. Functionally, cytoplasmic sequestration of BZR1 and RSG decreased expression levels of BR-responsive and GA-biosynthesis genes, respectively (Igarashi et al., 2001; Ishida et al., 2004; Gampala et al., 2007).

A role for 14-3-3 proteins in the cytoplasmic environment is also evident from protein-protein interaction and cytology studies. *In vitro* pull-down assays from developing *Arabidopsis* seeds using recombinant 14-3-3 χ or ε as bait revealed that ~40% of identified proteins were cytoplasmic (Swatek et al., 2011). Furthermore, 14-3-3 ω and φ/GFP fusion proteins were observed to be distributed throughout the cytoplasm of *Arabidopsis* trichomes and guard cells (Paul et al., 2005). A recent study demonstrated the light-dependent phosphorylation of *Arabidopsis* cytosolic invertase (CINV1) was followed by subsequent binding of 14-3-3 proteins, which enhanced the activity of invertase in root tissue (Gao et al., 2014). Collectively, these data suggest a strong role for 14-3-3 proteins in the cytoplasmic protein-protein interaction networks of plants.

#### Nucleus

As previously discussed, 14-3-3-mediated, transorganellar shuttling of transcription factors is a mechanism by which transcriptional activity can be repressed. Conversely, other reports have identified 14-3-3 proteins as transcriptional activators or co-activators. One of the first descriptions of enhanced transcriptional activity came from gene reporter assays in onion epithelial cells. In these assays, a 14-3-3 DNA-binding domain chimeric protein increased the transcriptional activity of a GAL4-GUS reporter gene (Pan et al., 1999). Additionally, 14-3-3 proteins can impact transcriptional activity by influencing multiprotein complex formation, as exemplified by the florigen activation complex (FAC). Co-expression of two components of the FAC, GF14b (a rice 14-3-3 paralog) and Hd3a [a rice FLOWERING LOCUST (FT) homolog], was largely cytosolic in rice protoplasts (Taoka et al., 2011). However, co-expression of a third component, OsFD1 (a bZIP transcription factor), completed the FAC, resulting in nuclear localization of all three components and increased transcript levels of OsMADS15 (an *Arabidopsis* APETALA1 homolog; Taoka et al., 2011). Despite a clear role of 14-3-3 proteins in nuclear-cytoplasmic shuttling, the mechanism by which they transition between compartments is currently debated. 14-3-3 proteins do contain a leucine-rich C-terminal helix, suggestive of a consensus nuclear export sequence that could control cytoplasmic sequestration of transcription factors (Lopez-Girona et al., 1999). Other evidence suggests that internal localization signals within 14-3-3 binding partners dictate subcellular movements (Brunet et al., 2002). Since evidence exists for both 14-3-3-mediated nuclear export and import, it is likely both mechanisms contribute to the localization of 14-3-3 proteins and their bindingpartners.

#### Chloroplast and mitochondria

Despite reported chloroplast and mitochondrial localization (Bunney et al., 2001; Sehnke et al., 2001; Ito et al., 2006), the mechanisms by which 14-3-3 proteins translocate the envelope membrane is currently unknown, as plant 14-3-3 proteins do not contain canonical N-terminal plastid or mitochondrial target peptides. It is possible 14-3-3 proteins “hitchhike” on precursor proteins during plastid or mitochondrial translocation; although, the precursor may not necessarily be the 14-3-3 binding partner *in organello*. For example, the target sequences of three tobacco chloroplast-bound precursor proteins, the small subunit of RuBisCO, and the oxygen-evolving complex subunits OE23 and OE33 contain a modified 14-3-3 binding motif (May and Soll, 2000). Site-directed mutagenesis of this consensus motif interferes with 14-3-3 and wheat germ lysate-synthesized precursor protein associations (May and Soll, 2000). Additionally, *in vivo* isolates of the ribulose-1,5-bisphosphate carboxylase/oxygenase small subunit precursor (preSSU) revealed a large oligomeric complex (~200 kDa) between preSSU, 14-3-3 proteins, and Hsp70 (May and Soll, 2000). This multimeric complex resulted in four-fold higher plastid translocation rates when compared to monomeric preSSU, suggesting 14-3-3 proteins actively participate in the mechanisms of precursor protein import pathways (May and Soll, 2000). Whether 14-3-3 mitochondrial or chloroplast import is a consequence of a “hitchhiking” mechanism remains unclear, but it should be considered since 14-3-3 proteins are directly involved in the import machinery of plastid precursor proteins (Figure 2).

#### Plasma membrane

The influence of 14-3-3 proteins extends beyond the regulation of soluble cytoplasmic proteins and into the realm of plasma membrane proteins. Proton (H^+^)-ATPases and potassium (K^+^) channels are two well-known plasma membrane 14-3-3 binding partners (Figure 2). Identification of the wilt-inducing phytotoxin fusicoccin receptor, a 14-3-3 protein, was a major advance in recognition of 14-3-3 associations with integral membrane proteins (Marra et al., 1994; Oecking et al., 1994). This finding facilitated the discovery of 14-3-3-mediated H^+^-ATPase activation, in which 14-3-3 binds to and alleviates the phosphorylated C-terminal auto-inhibitory domain (Fuglsang et al., 1999; Ottmann et al., 2007). H^+^-ATPase activation by 14-3-3 proteins also can be understood in the context of blue light-induced stomatal opening. This process is controlled through an electrochemical gradient generated by H^+^ pumping and ensuing K^+^ accumulation in guard cells (Assmann, 1993). Both blue light-induced H^+^-ATPase activation and 14-3-3/H^+^-ATPase interactions suggest a direct role of 14-3-3 proteins in the regulation of membrane potentials (Kinoshita and Shimazaki, 1999). Indeed, 14-3-3 proteins also influence the activity of voltage-gated K^+^ channels. Patch clamp assays in *Xenopus* oocytes overexpressing K^+^ channel (KAT1) were conditionally activated upon injection of recombinant 14-3-3 proteins (Sottocornola et al., 2006). Inside-out macro patch and binding assays suggest activation of KAT1 was mediated through a direct association with 14-3-3 proteins and not as a consequence of auxiliary pathways (Sottocornola et al., 2006, 2008). These results provide strong evidence that 14-3-3 proteins are a positive regulator of plasma membrane-bound ion channels, but they also support a synergistic role between H^+^ and K^+^ transport systems, perhaps in the regulation of stomatal aperture.

#### Vacuolar membrane

Similar to the regulation of plasma membrane proteins, 14-3-3 proteins also influence K^+^ channels in the vacuolar membrane. The vacuolar two-pore K^+^ channel (TPK1) is a Ca^2+^-activated regulator of cytoplasmic potassium levels (Bihler et al., 2005; Latz et al., 2007). Similar to P-type H^+^-ATPases, 14-3-3 proteins associate and activate TPK1 through a canonical 14-3-3 binding motif (Latz et al., 2007, 2012). In accordance with TPK1's Ca^2+^ activation, multiple Ca^2+^-dependent protein kinases (CPKs) phosphorylate TPK1 *in vitro*, including the salt stress-activated CPK3 (Mehlmer et al., 2010; Latz et al., 2012). Furthermore, plants treated with sodium chloride display elevated levels of TPK1 phosphorylation (Latz et al., 2012). These results suggest that, under salt-stress conditions, CPK3 phosphorylates TPK1, promoting 14-3-3-mediated activation and potassium efflux out of the vacuole. This proposed regulatory mechanism relies on 14-3-3 proteins acting in a tripartite system to maintain ionic homeostasis within cells and suggests a functional role for 14-3-3/CPK interactions. Interestingly, preferential phosphorylation of 14-3-3 ε (Ser65) by CPK3 was previously described *in vitro* (Swatek et al., 2014). Perhaps salt-stress conditions promote 14-3-3 phosphorylation by CPK3, thereby creating an additional mechanism of TPK1 regulation. Collectively, these studies support the role of 14-3-3 proteins as positive regulators of both plasma and vacuolar membrane ion homeostasis.

### Spatiotemporal expression of 14-3-3 genes in arabidopsis

#### Seedlings

**Figure 3:**
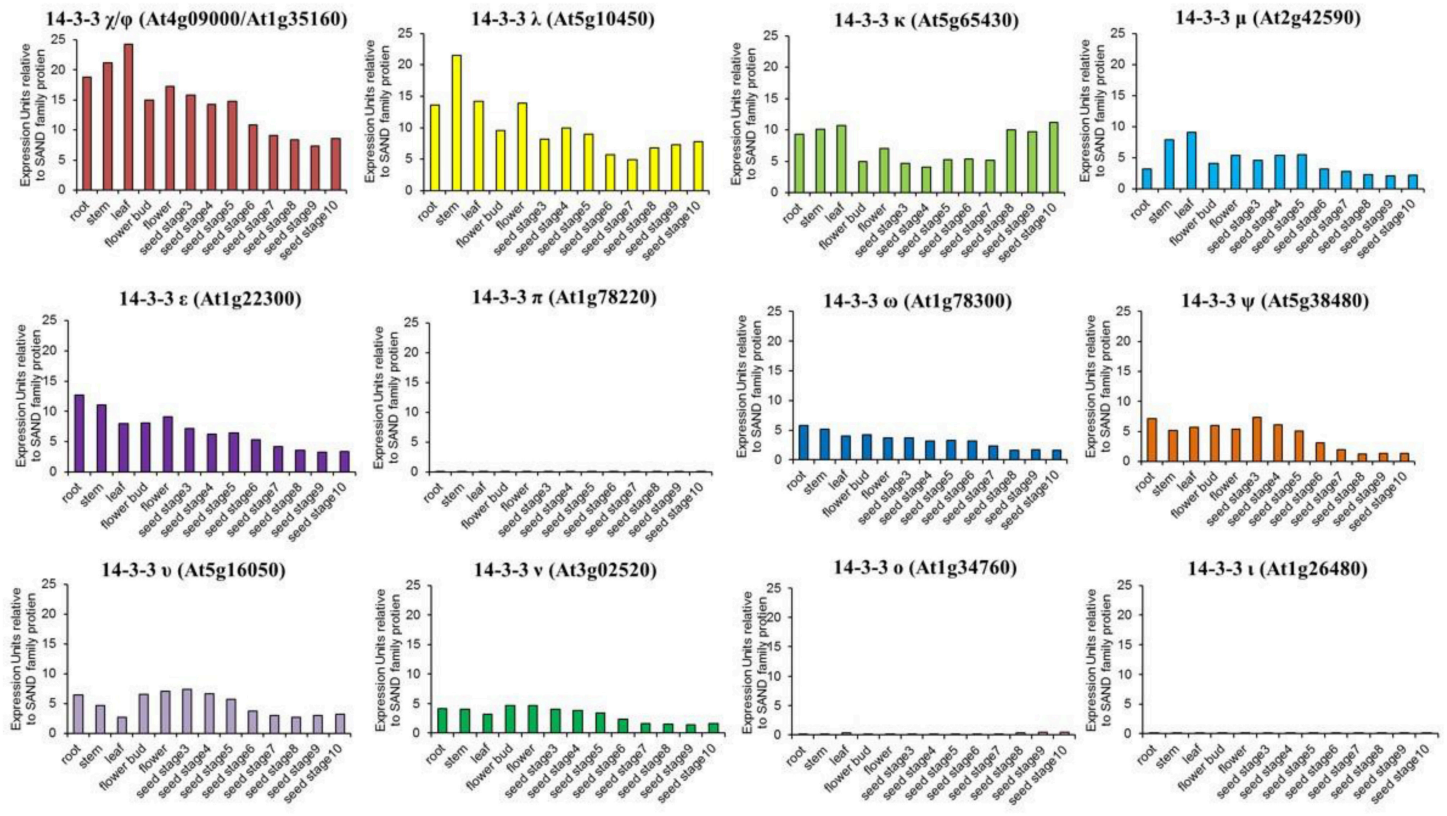
**Organ-specific *Arabidopsis* 14-3-3 expression profiles**. Gene expression data were obtained from EFP Browser. The y-axis represents raw expression units and is relative to SAND family (At2g28390) expression levels. The oligo-probe used in the microarrays cannot distinguish between 14-3-3 χ (At4g09000) and Φ (At1g35160), thus the gene expression data were plotted together. The following organs were used in the analysis: root, stem, leaf, flower bud, flower, seed stage3, seed stage4, seed stage5, seed stage6, seed stage8, seed stage9, and seed stage10.

The first description of 14-3-3 expression patterns in *Arabidopsis* was performed using 14-3-3 *upchi* promoter-driven GUS assays (Daugherty et al., 1996). GUS staining was primarily localized to the roots of developing seedlings but also was observed in flower buds, siliques, and the embryonic root of imbibed seeds (Daugherty et al., 1996). Subsequent microarray technologies allowed for a comprehensive analysis of 14-3-3 expression profiles (Schmid et al., 2005; Winter et al., 2007). Not only does the amplitude of 14-3-3 expression patterns vary considerably among isoforms, but the expression trends in different tissues are variable as well (Figure 3). These data imply that 14-3-3 isoforms are constitutively expressed to maintain, or respond to, the cellular demands of plants and also can become conditionally elevated.

Examination of 14-3-3 proteins in seedlings has revealed multiple biological roles, including involvement in primary metabolism, phototropism, and cold acclimation (Lancien and Roberts, 2006; Diaz et al., 2011; Sato et al., 2011). Of these, the role of 14-3-3 proteins in metabolism is best described. For instance, 14-3-3 χ protein levels become elevated when supplemented with glucose, creating a delay in the transition from heterotrophic to photoautotrophic growth (Sato et al., 2011). A molecular genetic analysis of 14-3-3 isoforms (κ, χ, ψ) using both overexpression and knockout lines revealed altered levels of metabolic intermediates of glycolysis, citric acid cycle, and shikimate pathways (Diaz et al., 2011). The altered metabolic intermediates in 14-3-3 overexpression lines were attributed to enzyme inhibition rather than activation; for example, phosphoenolpyruvate carboxylase (PEPCase) activity was reduced in 14-3-3 χ over-expression lines and unaltered in κ and ψ overexpression lines. A 14-3-3 χ association with PEPCase has been described in developing *Arabidopsis* seed (Swatek et al., 2011). A recent study used GUS assays and qPCR analysis to demonstrate enhanced expression of 14-3-3 ψ in seedling tissue exposed to low temperatures, implicating this isoform in freezing tolerance and cold acclimation (Catala et al., 2014). However, 14-3-3 ψ knockout seedlings displayed enhanced expression of cold-induced genes, suggesting 14-3-3 ψ is a negative regulator of cold-induced gene expression in *Arabidopsis* seedling tissue.

14-3-3 proteins also have mechanistic roles in the perception of light. 14-3-3 ν knockouts displayed impaired vertical hypocotyl growth, similar to phyA and phyB phytochrome mutants, suggesting that 14-3-3 ν is important in red light response (Mayfield et al., 2007). Interestingly, 14-3-3 μ knockout lines displayed no alterations in hypocotyl growth under the same conditions (Mayfield et al., 2007). Furthermore, yeast two-hybrid assays revealed interaction of 14-3-3 isoforms μ and ν with CONSTANS (CO), a transcriptional regulator with known involvement in the photoperiod pathway (Mayfield et al., 2007). Together, these phenotypes illustrate the importance of specific 14-3-3 isoforms in the control of both primary metabolism and phototropism in seedlings.

#### Leaves

**Figure 4:**
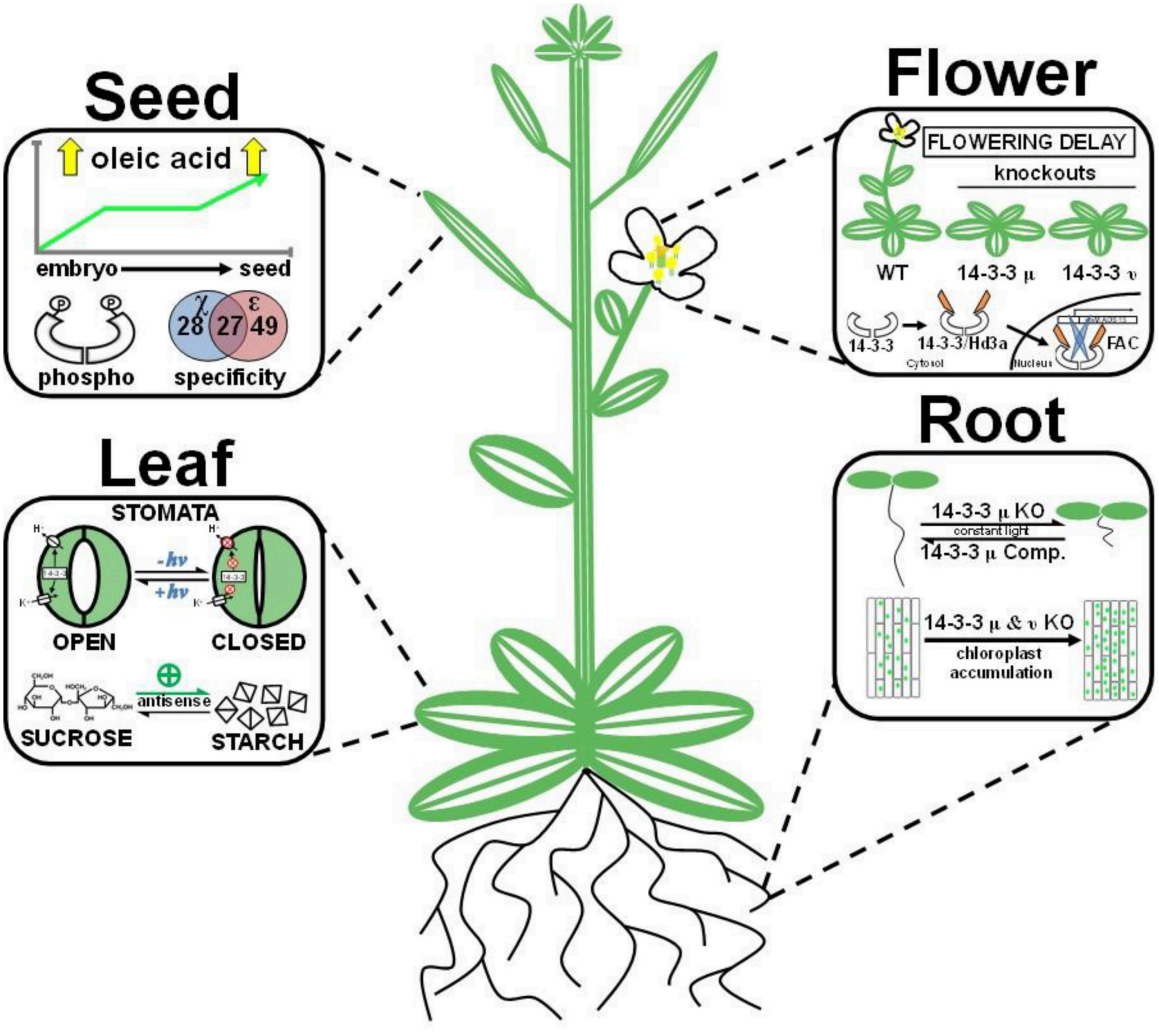
**Organ-specific mechanisms of plant 14-3-3 proteins**. The figure displays specific 14-3-3 functions across the seed, flower, leaf, and root. Seed: 14-3-3 proteins are phosphorylated, display isoform-specific interactions, and have elevated expression levels in an isogenic sunflower line bred for high oleic acid content. Flower: 14-3-3 proteins act as a positive regulator of floral development. 14-3-3 μ and ν knockouts result in a flowering delay. Additionally, rice 14-3-3 proteins create a heterohexamer florigen activation complex (FAC) with bZIP transcription factor OsFD1 and FLOWERING LOCUST (FT) homolog Hd3a, which induces expression of a gene (OsMADS15) involved in floral development. Leaf: 14-3-3 acts as a positive regulator of blue light (+*h*ν) induced stomata opening by activating P-type H^+^-ATPases and voltage-gated K^+^ channels. 14-3-3 antisense (ε and μ) lines increase starch accumulation in leaves. Root: 14-3-3 μ knockout lines (KO) severely stunt root growth, and complementation lines (Comp.) rescue the retarded root phenotype. 14-3-3 μ and ν knockout lines increase chloroplast accumulation in roots.

Blue light-induced stomatal opening through phototropins (PHOT1 and PHOT2) is one mechanism by which plants regulate stomatal aperture (Kinoshita and Shimazaki, 2001). PHOT2 and 14-3-3 λ have been shown to interact through a 14-3-3 recognition motif on the kinase domain of PHOT2 (Tseng and Briggs, 2010). Furthermore, a phot1/14-3-3 λ knockout line had decreased levels of stomatal opening upon increased blue light fluence rates, while phot2/14-3-3 λ and phot1/14-3-3 κ knockout lines had no effect (Tseng et al., 2012). Together, these data suggest that regulation of PHOT2-mediated stomatal opening is controlled through an isoform-specific interaction with 14-3-3 λ. Another component of blue light-induced stomatal opening is regulation of P-type H^+^-ATPases (Figure 4). As described above, P-type H^+^-ATPases are activated through 14-3-3 binding to the phosphorylated penultimate Thr of the autoinhibitory domain (Oecking et al., 1997; Kinoshita and Shimazaki, 1999, 2001; Alsterfjord et al., 2004; Ottmann et al., 2007).

14-3-3 proteins are implicated in regulation of leaf starch accumulation through light-dependent inactivation of enzymes, such as starch synthase. Immunolocalization of *Arabidopsis* 14-3-3 proteins visualized by electron microscopy revealed 14-3-3 immunodecorated chloroplast starch granules (Sehnke et al., 2001). In *Arabidopsis* leaf tissues, 14-3-3 ε and μ antisense lines increased leaf starch content up to four-fold higher than wild-type plants, suggesting these isoforms (both of the ε subgroup) negatively regulate starch accumulation (Figure 4; Sehnke et al., 2001). In addition, both biotinylated recombinant 14-3-3 overlay experiments and co-immunoprecipitation assays performed with maize starch proteins revealed 14-3-3 interactions with DU1 or DU1-like members of the starch synthase III family. Interestingly, leaf starch degradation pathways were unaffected.

The committed step of nitrogen metabolism, nitrate reductase, is controlled through a dark-dependent inactivation mechanism in mature *Spinacia oleracea* leaves, and this inhibition is strongly linked to nitrate reductase phosphorylation (Huber et al., 1992). Inactivation of nitrate reductase through phosphorylation and subsequent 14-3-3 binding is well-established in plants (Bachmann et al., 1996; Lambeck et al., 2010). Similar to seedlings, these studies reiterate the relationship between 14-3-3 proteins in phototropic responses and primary metabolism and suggest that 14-3-3 proteins negatively affect the pathways of carbon and nitrogen metabolism by preventing enzymatic activity.

#### Roots

The function of 14-3-3 isoforms in root development is conditional and isoform-specific. The most obvious example comes from 14-3-3 μ knockout lines, which have a ~75% reduction in root length when grown under constant light (Figure 4; Mayfield et al., 2012). Interestingly, 14-3-3 μ and ν knockout lines display increased chloroplast accumulation within roots when grown under constant white light (Figure 4; Mayfield et al., 2012). However, even this phenotype is conditional, with only the 14-3-3 μ lines displaying this phenotype under blue light conditions (Mayfield et al., 2012). An additional example comes from *Bradyrhizobium japonicum*-induced soybean nodulation. It was initially reported that transcript levels of soybean 14-3-3 SGF14c (the *Arabidopsis* 14-3-3 μ ortholog) become elevated in response to rhizobium-inoculation of soybeans (Brechenmacher et al., 2008). Recently, a functional role for 14-3-3 proteins in soybean nodulation has been described from RNA-mediated silencing of SGF14c and SGF14l (Radwan et al., 2012). RNA interference of these two 14-3-3 paralogs impaired both nodule development and maturation; while immature nodule formation increased by more than 20-fold, the total number of mature nodules was reduced three-fold (Radwan et al., 2012). Interestingly, this phenotype was specific, as no additional morphological changes in root anatomy were observed (Radwan et al., 2012). A recent study demonstrated involvement of *Arabidopsis* 14-3-3 μ in root growth responses under mild water stress conditions (He et al., 2015). After treatment with polyethylene glycol, 14-3-3 μ over-expression lines displayed increased root growth and root proton extrusion as well as enhanced allocation of carbon from the shoots to the roots when compared to wild-type. Another recent study performed genetic crosses of *Arabidopsis* T-DNA lines for six different 14-3-3 genes and assessed seedling primary root lengths (van Kleeff et al., 2014). Interestingly, the phenotypic analyses revealed decreased primary root lengths in the triple and quadruple mutants and no apparent difference in primary root lengths in single and double mutants. These results suggest involvement of multiple 14-3-3 isoforms in primary root growth and support previous reports of functional redundancy among 14-3-3 proteins. Collectively, these findings represent a growing trend of 14-3-3 paralogs in development-specific phenotypes.

#### Flowers

The initiation of floral development is the result of numerous biological inputs that converge to activate the reproductive phase of a plant's life cycle. Several studies have suggested 14-3-3 proteins are directly involved in floral development. Promoter-driven GUS reporter assays initially demonstrated expression of 14-3-3 χ in multiple flower organs (i.e., petals, pistils, and stems). Later, 14-3-3 μ and ν knockout lines were shown to negatively affect photoperiodic flowering under long day and short night conditions (**Figure 4;** Mayfield et al., 2007). However, plants grown under short day and long night conditions displayed no visual delay in floral development. A systematic phosphoproteomic screen of mature dehydrated pollen grains identified 609 phosphorylation sites (Mayank et al., 2012), a significant portion of which contain 14-3-3 binding motifs, suggesting a role for 14-3-3 proteins in pollen development (Mayank et al., 2012). Finally, as discussed above, 14-3-3 proteins are essential to the formation of a ternary FAC, which stimulates transcription of a floral development gene that ultimately drives floral development (Taoka et al., 2011).

#### Developing seed

A global proteomic analysis of seed maturation identified multiple 14-3-3 isoforms in soybean (SGF14a, SGF14b, SGF14d, SGF14c), rapeseed (ν, κ, ε), castor (ω, λ, ν, μ, o), and *Arabidopsis* (χ, ω, λ, $\ddot{\upsilon }$, κ, μ, ε) (Hajduch et al., 2005; Houston et al., 2009). In *Arabidopsis*, these isoforms collectively represent as much as 1% of the entire developing seed proteome from 2D gel analyses (Hajduch et al., 2010). A global phosphoproteomic profiling study of *Brassica napus* seed identified two phosphorylated 14-3-3 isoforms (χ and ε), suggesting 14-3-3 phosphorylation could influence seed development (Agrawal and Thelen, 2006). In a recent study performed in developing maize kernels, 77 specific binding partners were identified using affinity chromatography coupled to mass spectrometry for two 14-3-3 isoforms (Dou et al., 2015). Sixty percent of these binding partners were common to both isoforms, and many were implicated in various cellular processes, including protein destination and storage. As further evidence of 14-3-3 proteins role in seed oil accumulation, a 2D-DIGE-based proteomic analysis of a near-isogenic sunflower line bred for high oleic acid content displayed elevated levels of 14-3-3 protein expression compared to a parental line (Figure 4; Hajduch et al., 2007). A separate study performed in developing *Arabidopsis* seed used pull-down assays with recombinant 14-3-3 χ and ε as bait to identify 104 14-3-3 binding partners, 45 of which were functionally related to metabolism (Swatek et al., 2011). This comparative 14-3-3 interactome study revealed unique binding partner preferences between 14-3-3 χ and ε (Figure 4). For example, 14-3-3 χ preferentially forms hetero-dimers with phylogenetically similar 14-3-3 isoforms (ω, φ, ψ, ν) (Swatek et al., 2011). Collectively, these studies suggest a role of 14-3-3 proteins in storage reserve deposition during seed maturation.

### Discovery and function of 14-3-3 phosphorylation

Since 14-3-3 proteins are phosphorylated, this post-translational modification (PTM) increases the number of protein variants in a potentially functional manner. The first description of plant 14-3-3 phosphorylation came from a systematic study of phosphoproteins in developing seed of *B. napus* (Agrawal and Thelen, 2006). Using high-resolution 2D gels coupled to total and phosphoprotein multiplexed staining, it was discovered that 14-3-3 χ and ε are both phosphorylated (Agrawal and Thelen, 2006). Later, another phosphoproteomic study comparing knockout and overexpression lines of SnRK2.8 revealed differential phosphorylation of 14-3-3 κ and χ in *Arabidopsis* root tissues (Shin et al., 2007). In total,17 *in vivo* 14-3-3 phosphorylation sites on eight unique isoforms have been described (Benschop et al., 2007; Sugiyama et al., 2008; Jones et al., 2009; Reiland et al., 2009; Nakagami et al., 2010).

Further characterization of 14-3-3 phosphorylation through *in vitro* kinase assays revealed candidate protein kinases for *in vivo* phosphorylation sites. For instance, SnRK2.8 phosphorylated 14-3-3 κ and χ at Ser93 and Ser95, respectively. Additionally, multiple sites on 14-3-3 χ (Ser72, Ser88, Ser125, Thr156) and ε (Thr18, Ser65, Thr244) were phosphorylated by CPKs (CPK1, 3, 6, 8, 24, 28), none of which overlapped with SnRK2.8 (Swatek et al., 2014). Furthermore, one phosphorylation site, ε-Thr18, was unique to this isoform.

Despite *in vitro* and *in vivo* evidence of 14-3-3 phosphorylation, only recently has a functional role begun to emerge. In one study, 14-3-3 phosphorylation disrupted an association between 14-3-3 and CPK3, facilitating proteasomal degradation of CPK3 (Lachaud et al., 2013). Site-directed mutagenesis of a 14-3-3 phosphorylation site on 14-3-3 χ (Ser72 to Asp) prevented inhibition of nitrate reductase (Swatek et al., 2014). Similarly, phosphorylation of 14-3-3 ω at Ser62 and Ser67 caused destabilization of dimer formation *in vitro* (Denison et al., 2014). Interestingly, Ser67 of 14-3-3 ω aligns with Ser72 of 14-3-3 χ, and this residue is conserved across all isoforms. Collectively, these data suggest a general mechanism of functional phosphorylation for the *Arabidopsis* 14-3-3 isoforms, specifically by reducing the affinity for binding partner interactions possibly through dimer destabilization.

Although, our understanding of 14-3-3 phosphorylation is limited, evidence suggests phosphorylation of semi-conserved residues by unique kinases could contribute to their functional diversity in plants. While additional PTMs on plant 14-3-3 proteins have been identified, including ubiquitination and Lys-acetylation, their functional significance is even less clear than phosphorylation, though speculation is centered on proteasomal degradation pathways (Sehnke et al., 2001; Fuller et al., 2006).

## Concluding remarks

This review highlighted recent advances in the plant 14-3-3 field regarding regulation at the spatiotemporal and subcellular levels as well as post-translational modification. The large number of isoforms expressed in plants, coupled to their redundant involvement in an intricate web of interactions, reveals the complexity of plant 14-3-3 research at present. At the cellular level, 14-3-3 proteins regulate the localization and function of their binding partners by means of physical occlusion, scaffolding, or altered conformational changes. While the immediate impact of these protein associations is often clear, their downstream effects are frequently ambiguous. In tissues, isoform-specific 14-3-3 knockout and overexpression can visually affect specific developmental stages. However, these phenotypes are often conditional and subtle, which is likely a result of 14-3-3 functional redundancy. Furthermore, discovery of 14-3-3 phosphorylation presents an additional area of future focus, as several studies have identified 14-3-3 phosphorylation sites *in planta*. Currently, the known effects of 14-3-3 phosphorylation are few and include altered monomer/dimer ratios and disruption of binding partner interactions. The need for further investigation is apparent, as the effects of this modification on 14-3-3 regulation and function appear to be substantial.

## Author contributions

RW, KS, and JT wrote the review. RW and KS designed the figures for the review.

### Conflict of interest statement

The authors declare that the research was conducted in the absence of any commercial or financial relationships that could be construed as a potential conflict of interest.

## Abbreviations

PTM: post-translational modification
CPK: calcium-dependent protein kinase
RSG: repression of shoot growth
GA: gibberellic acid
FAC: florigen activation complex
NES: nuclear export sequence
GUS: β-glucuronidase
T-DNA: transfer DNA
NR: nitrate reductase
TF: transcription factor.
